# Supplementary material for: The causal relationship between the human gut microbiota and pyogenic arthritis: a Mendelian randomization study
Source: Front Cell Infect Microbiol. 2024 Nov 26;14:1452480. doi: 10.3389/fcimb.2024.1452480 (PMC11629706; doi:10.3389/fcimb.2024.1452480)
Supplement: Supplementary file 1 [file DataSheet1.zip › mendelian test/ebi-a-GCST90027819.csv_leave_one_out.pdf]

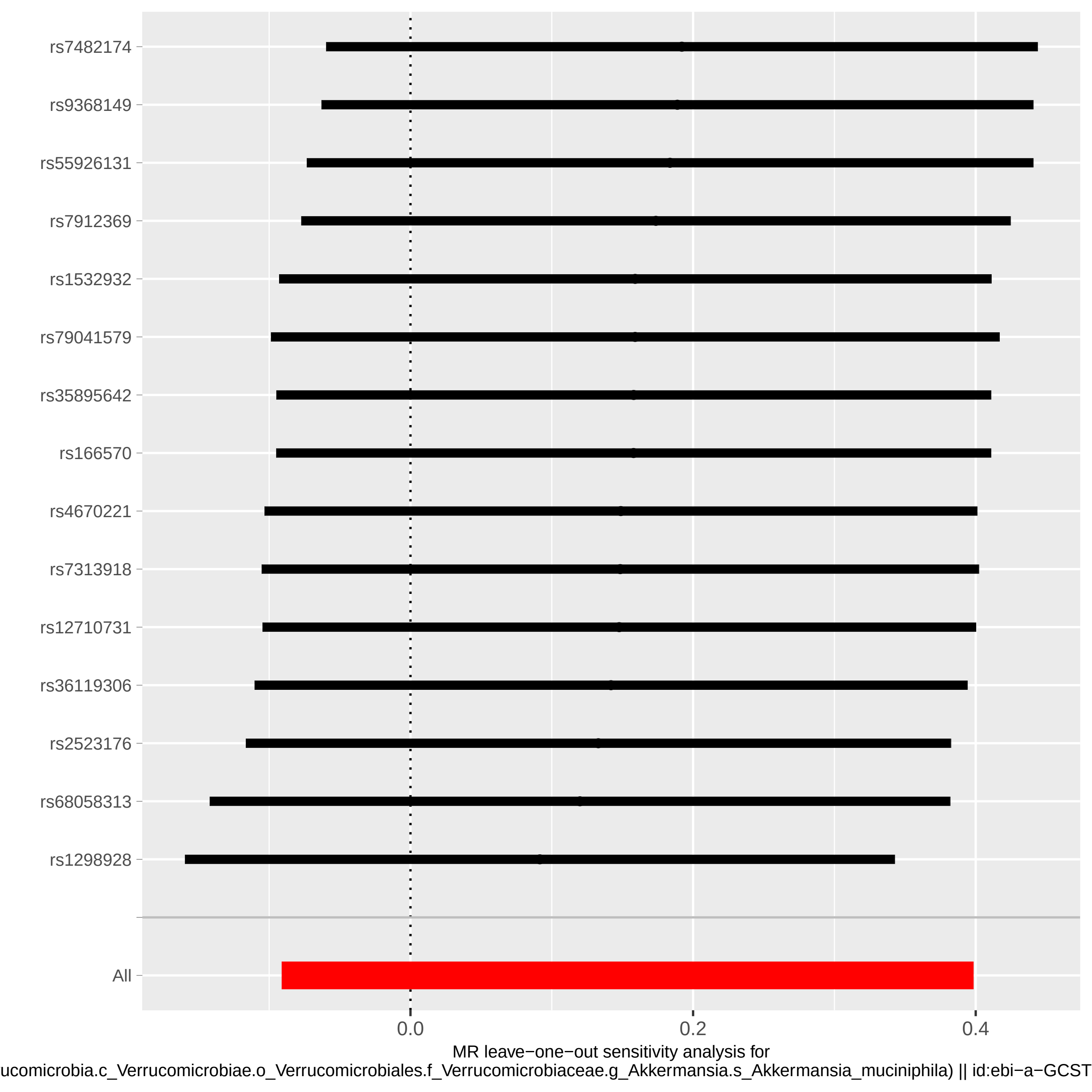
rs7482174
rs9368149
rs55926131
rs7912369
rs1532932
rs79041579
rs35895642
rs166570
rs4670221
rs7313918
rs12710731
rs36119306
rs2523176
rs68058313
rs1298928
All
0.0
0.2
0.4
MR leave−one−out sensitivity analysis for
ucomicrobia.c_Verrucomicrobiae.o_Verrucomicrobiales.f_Verrucomicrobiaceae.g_Akkermansia.s_Akkermansia_muciniphila) || id:ebi−a−GCST
